# Phen­yl(1-phenyl­sulfonyl-1*H*-indol-2-yl)methanone

**DOI:** 10.1107/S1600536811008439

**Published:** 2011-03-12

**Authors:** S. Ranjith, A. SubbiahPandi, E. Govindan, V. Dhayalan, A. K. MohanaKrishnan

**Affiliations:** a Department of Physics, Presidency College (Autonomous), Chennai 600 005, India; b Department of Organic Chemistry, University of Madras, Guindy Campus, Chennai 600 025, India

## Abstract

The asymmetric unit of the title compound, C_21_H_15_NO_3_S, contains two crystallographically independent mol­ecules. As a result of the electron-withdrawing character of the phenyl­sulfonyl groups, the N—C*sp* ^2^ bond lengths are slightly longer than the anti­cipated value of approximately 1.35 Å for N atoms with planar configurations. Both unique S atoms have a distorted tetra­hedral configuration. In each mol­ecule, the indole ring system is essentially planar (r.m.s. deviations for all non-H atoms of 0.020 and 0.023 Å). In one mol­ecule, the indole ring system makes dihedral angles of 65.7 (8) and 73.4 (8)°, respectively, with the benzene and phenyl rings [62.2 (7) and 72.1 (7)°, respectively, in the other mol­ecule].

## Related literature

For the biological activity of compounds containing an indole ring system, see: Ma *et al.* (2001 ▶); Zhou *et al.* (2006 ▶); Zhao *et al.* (2002 ▶); Williams *et al.* (1993 ▶). For related structures, see: Chakkaravarthi *et al.* (2010 ▶); Kavitha *et al.* (2010 ▶). For a discussion of the geometry at the N atom, see: Beddoes *et al.* (1986 ▶). For standard bond-length data, see: Allen *et al.* (1987 ▶).
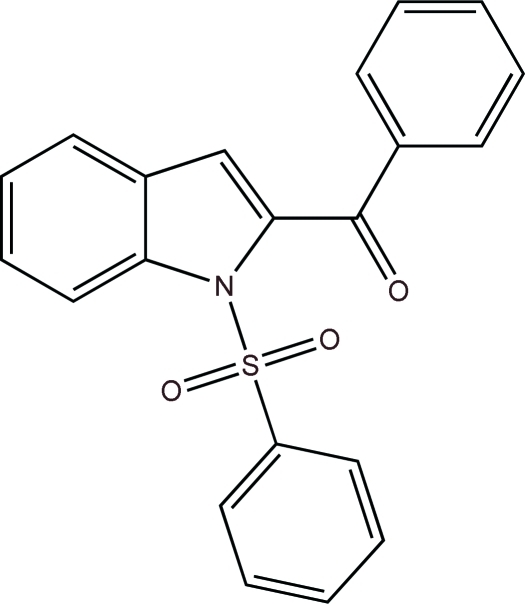


## Experimental

### 

#### Crystal data

C_21_H_15_NO_3_S
*M* *_r_* = 361.40
Triclinic, 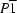

*a* = 9.3291 (6) Å
*b* = 11.1498 (7) Å
*c* = 17.8134 (10) Å
α = 89.433 (3)°
β = 81.623 (2)°
γ = 71.993 (3)°
*V* = 1742.13 (18) Å^3^
*Z* = 4
Mo *K*α radiation
μ = 0.21 mm^−1^
*T* = 293 K
0.25 × 0.22 × 0.19 mm

#### Data collection

Bruker APEXII CCD area-detector diffractometer
Absorption correction: multi-scan (*SADABS*; Sheldrick, 1996 ▶) *T*_min_ = 0.981, *T*_max_ = 0.985
30464 measured reflections
8730 independent reflections
6363 reflections with *I* > 2σ(*I*)
*R*_int_ = 0.041

#### Refinement

*R*[*F* ^2^ > 2σ(*F* ^2^)] = 0.040
*wR*(*F* ^2^) = 0.138
*S* = 0.99
8730 reflections
470 parameters
H-atom parameters constrained
Δρ_max_ = 0.27 e Å^−3^
Δρ_min_ = −0.33 e Å^−3^

### 

Data collection: *APEX2* (Bruker, 2007 ▶); cell refinement: *SAINT* (Bruker, 2007 ▶); data reduction: *SAINT* program(s) used to solve structure: *SHELXTL* (Sheldrick, 2008 ▶); program(s) used to refine structure: *SHELXTL*; molecular graphics: *ORTEP-3* (Farrugia, 1997 ▶); software used to prepare material for publication: *SHELXTL* and *PLATON* (Spek, 2009 ▶).

## Supplementary Material

Crystal structure: contains datablocks global, I. DOI: 10.1107/S1600536811008439/nk2081sup1.cif

Structure factors: contains datablocks I. DOI: 10.1107/S1600536811008439/nk2081Isup2.hkl

Additional supplementary materials:  crystallographic information; 3D view; checkCIF report

## Figures and Tables

**Fig. 1.**
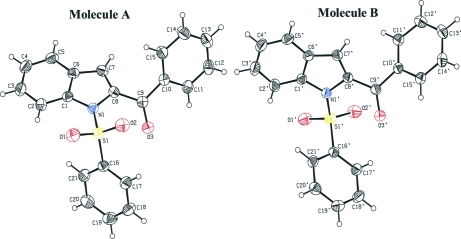
The two crystallographically unique molecules. Displacement ellipsoids are drawn at the 30% probability level.

## supplementary crystallographic information

### Comment

The indole ring system is present in a number of natural products, many of which are found to possess anticancer, antimalarial and antihypertensive activities (Ma *et al.*, 2001; Zhou *et al.*, 2006; Zhao *et al.*, 2002). In addition, phenylsulfonyl indole compounds inhibit the HIV-1 RT enzyme *in vitro* and HTLVIIIb viral spread in MT-4 human T-lymphoid cells (Williams *et al.*, 1993). Sulfonamide derivates are well known drugs and are used to control diseases caused by bacterial infections. Against this background and in order to obtain detailed information on molecular conformations in the solid state, an X-ray study of the title compound was carried out.

X-Ray analysis confirms the molecular structure and atom connectivity as illustrated in Fig. 1. The bond lengths and angles in (Fig. 1) agree with those observed in other phenylsulfonylindoles (Chakkaravarthi *et al.*, 2010). In both the molecules, the indole ring systems are essentially planar, with r.m.s. deviation of 0.028 and 0.034Å for atoms C6 and C6'. In both the molecules, the sum of bond angles around N1[359.1 (11)°] of the pyrrole ring is in accordance with *sp*^3^ hybridization (Beddoes *et al.*, 1986). In molecule A, indole ring system make the dihedral angles of 65.7 (8) and 73.4 (8)°, respectively, with the benzene and phenyl rings and also in molecule B, indole ring system make the dihedral angles of 62.2 (7) and 72.1 (7)°, respectively, with the benzene and phenyl rings.

The S–O, S–C, and S–N distances are 1.421 (1), 1.755 (2) and 1.673 (1) Å, respectively and these values are comparable as observed in similar structures (Chakkaravarthi *et al.*, 2010). As a result of the electron-withdrawing character of the phenylsulfonyl groups, the N–C*sp*^2^ bond lengths, *viz*. N1–C1 [1.414 (2) Å], N1'–C1' [1.415 (2) Å], N1–C8 [1.416 (2) Å] and N1'–C8' [1.417 (2) Å], are longer than the mean value of 1.355 (1)Å reported for N atoms with planar configurations (Allen *et al.*, 1987). In both the molecules,the S atom exhibits significant deviation from that of a regular tetrahedron, with the largest deviations being seen for the O–S–O [O1–S1–O2 119.5 (9)° & O1'–S1'–O2' 119.7 (8)°] and O–S–N angles [O1–S1–N1 105.1 (7)° & O1'–S1'–N1' 104.8 (7)°]. The widening of the angles may be due to repulsive interactions between the two short S=O bonds, similar to what is observed in related structures (Kavitha *et al.*, 2010).

### Experimental

To a solution of *N*-(2-Formylphenyl)benzenesulfonamide (0.5 g, 1.91 mmol) in dry CH_3_CN (20 ml), K_2_CO_3_ (0.8 g, 5.79 mmol), phenacylbromides (0.45 g, 2.26 mmol) were added. The reaction mixture was stirred at room temperature for 6 h under N_2_ atmosphere. The solvent was removed and the residue was quenched with ice-water (50 ml), extracted with chloroform (3 *x* 10 ml) and dried (Na_2_SO_4_). Removal of solvent followed by the residue was dissolved in CH_3_CN (20 ml), Conc.HCl (3 ml) was added. The reaction mixture was then refluxed for 2 h. It was then poured over ice-water (50 ml), extracted with CHCl_3_ (3 *x* 10 ml) and dried (Na_2_SO_4_). Single crystals suitable for X-ray diffraction were obtained by slow evaporation of a solution of the title compound in methanol at room temperature.

### Refinement

All H atoms were fixed geometrically and allowed to ride on their parent C atoms, with C—H distances fixed in the range 0.93–0.97 Å with *U*_iso_(H) = 1.5*U*_eq_(C) for methyl H 1.2*U*_eq_(C) for other H atoms.

### Crystal data

**Table:**

C_21_H_15_NO_3_S	*Z* = 4
*M**_r_* = 361.40	*F*(000) = 752
Triclinic, *P*1	*D*_x_ = 1.378 Mg m^−^^3^
Hall symbol: -P 1	Mo *K*α radiation, λ = 0.71073 Å
*a* = 9.3291 (6) Å	Cell parameters from 8730 reflections
*b* = 11.1498 (7) Å	θ = 1.2–28.7°
*c* = 17.8134 (10) Å	µ = 0.21 mm^−^^1^
α = 89.433 (3)°	*T* = 293 K
β = 81.623 (2)°	Block, white
γ = 71.993 (3)°	0.25 × 0.22 × 0.19 mm
*V* = 1742.13 (18) Å^3^	

### Data collection

**Table:**

Bruker APEXII CCD area-detector diffractometer	8730 independent reflections
Radiation source: fine-focus sealed tube	6363 reflections with *I* > 2σ(*I*)
graphite	*R*_int_ = 0.041
ω and φ scans	θ_max_ = 28.7°, θ_min_ = 1.2°
Absorption correction: multi-scan (*SADABS*; Sheldrick, 1996)	*h* = −12→12
*T*_min_ = 0.981, *T*_max_ = 0.985	*k* = −14→14
30464 measured reflections	*l* = −23→24

### Refinement

**Table:**

Refinement on *F*^2^	Secondary atom site location: difference Fourier map
Least-squares matrix: full	Hydrogen site location: inferred from neighbouring sites
*R*[*F*^2^ > 2σ(*F*^2^)] = 0.040	H-atom parameters constrained
*wR*(*F*^2^) = 0.138	*w* = 1/[σ^2^(*F*_o_^2^) + (0.082*P*)^2^ + 0.1847*P*] where *P* = (*F*_o_^2^ + 2*F*_c_^2^)/3
*S* = 0.99	(Δ/σ)_max_ = 0.001
8730 reflections	Δρ_max_ = 0.27 e Å^−^^3^
470 parameters	Δρ_min_ = −0.33 e Å^−^^3^
0 restraints	Extinction correction: *SHELXTL* (Sheldrick, 2008), Fc^*^=kFc[1+0.001xFc^2^λ^3^/sin(2θ)]^-1/4^
Primary atom site location: structure-invariant direct methods	Extinction coefficient: 0.085 (3)

### Special details

**Table:**

Geometry. All e.s.d.'s (except the e.s.d. in the dihedral angle between two l.s. planes) are estimated using the full covariance matrix. The cell e.s.d.'s are taken into account individually in the estimation of e.s.d.'s in distances, angles and torsion angles; correlations between e.s.d.'s in cell parameters are only used when they are defined by crystal symmetry. An approximate (isotropic) treatment of cell e.s.d.'s is used for estimating e.s.d.'s involving l.s. planes.
Refinement. Refinement of *F*^2^ against ALL reflections. The weighted *R*-factor *wR* and goodness of fit *S* are based on *F*^2^, conventional *R*-factors *R* are based on *F*, with *F* set to zero for negative *F*^2^. The threshold expression of *F*^2^ > σ(*F*^2^) is used only for calculating *R*-factors(gt) *etc*. and is not relevant to the choice of reflections for refinement. *R*-factors based on *F*^2^ are statistically about twice as large as those based on *F*, and *R*- factors based on ALL data will be even larger.

### Fractional atomic coordinates and isotropic or equivalent isotropic displacement parameters (Å^2^)

**Table:**

	*x*	*y*	*z*	*U*_iso_*/*U*_eq_	
C1	1.12210 (18)	0.80297 (15)	0.33999 (9)	0.0466 (4)	
C1'	1.04606 (17)	0.31674 (14)	0.15061 (8)	0.0427 (3)	
C2'	1.1137 (2)	0.19223 (16)	0.16963 (10)	0.0549 (4)	
H2'	1.0634	0.1321	0.1694	0.066*	
C2	1.1947 (2)	0.67756 (17)	0.31637 (11)	0.0593 (4)	
H2	1.1406	0.6201	0.3160	0.071*	
C3	1.3500 (2)	0.6420 (2)	0.29356 (11)	0.0679 (5)	
H3	1.4015	0.5585	0.2779	0.082*	
C3'	1.2583 (2)	0.16248 (19)	0.18871 (10)	0.0620 (5)	
H3'	1.3061	0.0802	0.2016	0.074*	
C4'	1.3354 (2)	0.25122 (19)	0.18944 (10)	0.0630 (5)	
H4'	1.4333	0.2271	0.2022	0.076*	
C4	1.4318 (2)	0.7265 (2)	0.29317 (12)	0.0701 (6)	
H4	1.5367	0.6987	0.2777	0.084*	
C5	1.3603 (2)	0.8508 (2)	0.31527 (11)	0.0621 (5)	
H5	1.4157	0.9074	0.3146	0.074*	
C5'	1.26861 (18)	0.37364 (18)	0.17157 (10)	0.0559 (4)	
H5'	1.3200	0.4330	0.1720	0.067*	
C6	1.20280 (18)	0.89080 (16)	0.33877 (9)	0.0486 (4)	
C6'	1.12073 (17)	0.40739 (15)	0.15260 (9)	0.0454 (3)	
C7	1.09697 (19)	1.00924 (16)	0.36680 (10)	0.0514 (4)	
H7	1.1201	1.0840	0.3712	0.062*	
C7'	1.02433 (17)	0.52315 (15)	0.12936 (9)	0.0475 (4)	
H7'	1.0458	0.5993	0.1265	0.057*	
C8'	0.89647 (17)	0.50409 (14)	0.11210 (9)	0.0429 (3)	
C8	0.95772 (18)	0.99525 (15)	0.38603 (9)	0.0461 (3)	
C9'	0.78298 (17)	0.58882 (14)	0.06981 (9)	0.0439 (3)	
C9	0.82428 (18)	1.08384 (16)	0.43345 (9)	0.0493 (4)	
C10'	0.74687 (16)	0.72726 (14)	0.08203 (9)	0.0415 (3)	
C10	0.79218 (18)	1.22132 (15)	0.42177 (9)	0.0468 (4)	
C11	0.72410 (19)	1.30446 (17)	0.48355 (10)	0.0537 (4)	
H11	0.7030	1.2735	0.5312	0.064*	
C11'	0.74584 (19)	0.78266 (15)	0.15170 (9)	0.0493 (4)	
H11'	0.7738	0.7326	0.1925	0.059*	
C12'	0.7028 (2)	0.91320 (17)	0.16001 (11)	0.0586 (4)	
H12'	0.6983	0.9508	0.2070	0.070*	
C12	0.6877 (2)	1.43246 (18)	0.47447 (12)	0.0635 (5)	
H12	0.6452	1.4878	0.5163	0.076*	
C13	0.7142 (2)	1.47895 (18)	0.40350 (14)	0.0707 (5)	
H13	0.6873	1.5656	0.3974	0.085*	
C13'	0.6669 (2)	0.98698 (16)	0.09905 (12)	0.0595 (5)	
H13'	0.6400	1.0743	0.1047	0.071*	
C14'	0.67055 (19)	0.93190 (16)	0.02912 (11)	0.0556 (4)	
H14'	0.6486	0.9820	−0.0124	0.067*	
C14	0.7801 (3)	1.39750 (19)	0.34158 (13)	0.0721 (6)	
H14	0.7972	1.4293	0.2938	0.087*	
C15	0.8211 (2)	1.26808 (17)	0.35029 (10)	0.0593 (4)	
H15	0.8676	1.2130	0.3087	0.071*	
C15'	0.70660 (17)	0.80322 (15)	0.02128 (9)	0.0480 (4)	
H15'	0.7041	0.7666	−0.0250	0.058*	
C16	0.78168 (17)	0.77029 (15)	0.46542 (9)	0.0460 (3)	
C16'	0.76892 (17)	0.26929 (14)	0.03053 (9)	0.0425 (3)	
C17'	0.64230 (19)	0.31640 (17)	−0.00556 (10)	0.0525 (4)	
H17'	0.5545	0.3754	0.0191	0.063*	
C17	0.6358 (2)	0.81398 (18)	0.50410 (11)	0.0604 (4)	
H17	0.5582	0.8697	0.4820	0.073*	
C18'	0.6491 (2)	0.27381 (19)	−0.07907 (11)	0.0615 (5)	
H18'	0.5651	0.3047	−0.1042	0.074*	
C18	0.6068 (3)	0.7732 (2)	0.57678 (12)	0.0728 (6)	
H18	0.5088	0.8018	0.6039	0.087*	
C19	0.7221 (3)	0.6908 (2)	0.60898 (11)	0.0720 (6)	
H19	0.7017	0.6646	0.6580	0.086*	
C19'	0.7790 (2)	0.18625 (18)	−0.11532 (10)	0.0595 (4)	
H19'	0.7826	0.1588	−0.1649	0.071*	
C20	0.8674 (2)	0.64670 (19)	0.56934 (12)	0.0674 (5)	
H20	0.9447	0.5904	0.5913	0.081*	
C20'	0.9040 (2)	0.13900 (17)	−0.07847 (11)	0.0572 (4)	
H20'	0.9913	0.0793	−0.1031	0.069*	
C21'	0.89966 (18)	0.18017 (15)	−0.00517 (10)	0.0500 (4)	
H21'	0.9835	0.1485	0.0200	0.060*	
C21	0.8978 (2)	0.68629 (17)	0.49705 (10)	0.0559 (4)	
H21	0.9955	0.6568	0.4697	0.067*	
N1	0.96883 (15)	0.86837 (12)	0.36984 (7)	0.0467 (3)	
N1'	0.90578 (14)	0.37628 (11)	0.12457 (7)	0.0426 (3)	
O1'	0.80070 (16)	0.20614 (12)	0.16827 (7)	0.0645 (4)	
O1	0.87435 (16)	0.70347 (13)	0.32343 (7)	0.0672 (4)	
O2'	0.62388 (13)	0.41561 (13)	0.14936 (7)	0.0630 (3)	
O2	0.69510 (15)	0.91680 (14)	0.35443 (8)	0.0673 (4)	
O3	0.74722 (15)	1.04506 (12)	0.48283 (7)	0.0645 (3)	
O3'	0.72679 (15)	0.54592 (11)	0.02333 (7)	0.0602 (3)	
S1'	0.76114 (4)	0.31681 (4)	0.12514 (2)	0.04706 (13)	
S1	0.81966 (5)	0.81457 (4)	0.37162 (2)	0.05089 (13)	

### Atomic displacement parameters (Å^2^)

**Table:**

	*U*^11^	*U*^22^	*U*^33^	*U*^12^	*U*^13^	*U*^23^
C1	0.0480 (8)	0.0498 (8)	0.0411 (8)	−0.0155 (7)	−0.0035 (6)	0.0043 (7)
C1'	0.0429 (8)	0.0457 (8)	0.0392 (7)	−0.0125 (6)	−0.0073 (6)	0.0004 (6)
C2'	0.0607 (10)	0.0490 (9)	0.0533 (10)	−0.0134 (8)	−0.0115 (8)	0.0090 (7)
C2	0.0634 (11)	0.0521 (10)	0.0576 (10)	−0.0154 (8)	0.0006 (8)	−0.0032 (8)
C3	0.0633 (11)	0.0638 (11)	0.0618 (12)	−0.0044 (9)	0.0048 (9)	0.0003 (9)
C3'	0.0611 (11)	0.0620 (11)	0.0512 (10)	−0.0002 (9)	−0.0134 (8)	0.0085 (8)
C4'	0.0468 (9)	0.0795 (13)	0.0536 (10)	−0.0025 (9)	−0.0160 (8)	−0.0050 (9)
C4	0.0478 (10)	0.0844 (14)	0.0652 (12)	−0.0069 (10)	0.0018 (9)	0.0135 (11)
C5	0.0480 (9)	0.0751 (12)	0.0648 (11)	−0.0225 (9)	−0.0077 (8)	0.0190 (10)
C5'	0.0421 (8)	0.0696 (11)	0.0554 (10)	−0.0145 (8)	−0.0109 (7)	−0.0107 (8)
C6	0.0480 (8)	0.0543 (9)	0.0459 (8)	−0.0189 (7)	−0.0093 (7)	0.0106 (7)
C6'	0.0422 (8)	0.0493 (8)	0.0449 (8)	−0.0135 (7)	−0.0085 (6)	−0.0040 (7)
C7	0.0527 (9)	0.0491 (9)	0.0562 (10)	−0.0207 (7)	−0.0100 (7)	0.0083 (7)
C7'	0.0453 (8)	0.0435 (8)	0.0574 (9)	−0.0176 (7)	−0.0105 (7)	−0.0017 (7)
C8'	0.0432 (8)	0.0393 (7)	0.0479 (8)	−0.0146 (6)	−0.0084 (6)	0.0016 (6)
C8	0.0489 (8)	0.0442 (8)	0.0464 (8)	−0.0151 (7)	−0.0093 (7)	0.0042 (7)
C9'	0.0413 (8)	0.0439 (8)	0.0480 (8)	−0.0145 (6)	−0.0082 (6)	0.0016 (6)
C9	0.0495 (9)	0.0514 (9)	0.0483 (9)	−0.0169 (7)	−0.0092 (7)	0.0025 (7)
C10'	0.0366 (7)	0.0411 (7)	0.0469 (8)	−0.0125 (6)	−0.0054 (6)	0.0020 (6)
C10	0.0469 (8)	0.0469 (8)	0.0472 (9)	−0.0137 (7)	−0.0110 (7)	−0.0008 (7)
C11	0.0499 (9)	0.0575 (10)	0.0520 (9)	−0.0134 (8)	−0.0085 (7)	−0.0041 (8)
C11'	0.0521 (9)	0.0501 (9)	0.0459 (9)	−0.0169 (7)	−0.0061 (7)	0.0022 (7)
C12'	0.0617 (10)	0.0544 (10)	0.0585 (10)	−0.0194 (8)	−0.0012 (8)	−0.0122 (8)
C12	0.0565 (10)	0.0572 (11)	0.0749 (13)	−0.0128 (9)	−0.0129 (9)	−0.0175 (9)
C13	0.0742 (13)	0.0469 (10)	0.0918 (16)	−0.0160 (9)	−0.0213 (11)	0.0011 (10)
C13'	0.0523 (10)	0.0404 (8)	0.0825 (13)	−0.0135 (7)	−0.0015 (9)	0.0004 (9)
C14'	0.0475 (9)	0.0502 (9)	0.0667 (11)	−0.0122 (7)	−0.0084 (8)	0.0154 (8)
C14	0.0906 (15)	0.0569 (11)	0.0682 (12)	−0.0203 (10)	−0.0163 (11)	0.0143 (10)
C15	0.0727 (12)	0.0522 (10)	0.0505 (10)	−0.0154 (9)	−0.0103 (9)	0.0018 (8)
C15'	0.0441 (8)	0.0505 (9)	0.0480 (9)	−0.0125 (7)	−0.0075 (7)	0.0042 (7)
C16	0.0454 (8)	0.0494 (8)	0.0485 (9)	−0.0218 (7)	−0.0079 (7)	−0.0002 (7)
C16'	0.0414 (7)	0.0425 (8)	0.0492 (8)	−0.0202 (6)	−0.0090 (6)	0.0036 (6)
C17'	0.0431 (8)	0.0557 (9)	0.0591 (10)	−0.0130 (7)	−0.0135 (7)	−0.0017 (8)
C17	0.0479 (9)	0.0646 (11)	0.0661 (11)	−0.0161 (8)	−0.0030 (8)	0.0049 (9)
C18'	0.0569 (10)	0.0734 (12)	0.0590 (11)	−0.0206 (9)	−0.0234 (8)	0.0028 (9)
C18	0.0672 (12)	0.0823 (14)	0.0642 (12)	−0.0260 (11)	0.0116 (10)	−0.0008 (11)
C19	0.0952 (16)	0.0792 (14)	0.0492 (10)	−0.0406 (12)	−0.0055 (10)	0.0034 (10)
C19'	0.0677 (11)	0.0671 (11)	0.0502 (10)	−0.0294 (9)	−0.0108 (8)	−0.0017 (8)
C20	0.0764 (13)	0.0684 (12)	0.0638 (12)	−0.0252 (10)	−0.0255 (10)	0.0125 (10)
C20'	0.0509 (9)	0.0571 (10)	0.0606 (11)	−0.0166 (8)	0.0010 (8)	−0.0061 (8)
C21'	0.0417 (8)	0.0506 (9)	0.0590 (10)	−0.0143 (7)	−0.0116 (7)	0.0040 (7)
C21	0.0479 (9)	0.0632 (10)	0.0577 (10)	−0.0182 (8)	−0.0090 (8)	0.0008 (8)
N1	0.0466 (7)	0.0470 (7)	0.0483 (7)	−0.0194 (6)	−0.0016 (6)	−0.0014 (6)
N1'	0.0433 (7)	0.0406 (6)	0.0490 (7)	−0.0177 (5)	−0.0130 (5)	0.0048 (5)
O1'	0.0880 (9)	0.0694 (8)	0.0565 (7)	−0.0497 (7)	−0.0219 (7)	0.0190 (6)
O1	0.0812 (9)	0.0753 (9)	0.0569 (7)	−0.0428 (7)	−0.0059 (6)	−0.0125 (6)
O2'	0.0451 (6)	0.0798 (9)	0.0639 (8)	−0.0232 (6)	0.0018 (6)	−0.0145 (7)
O2	0.0564 (7)	0.0859 (9)	0.0659 (8)	−0.0249 (7)	−0.0245 (6)	0.0188 (7)
O3	0.0687 (8)	0.0578 (7)	0.0618 (8)	−0.0197 (6)	0.0071 (6)	0.0036 (6)
O3'	0.0687 (8)	0.0488 (7)	0.0688 (8)	−0.0171 (6)	−0.0303 (6)	−0.0006 (6)
S1'	0.0479 (2)	0.0545 (2)	0.0467 (2)	−0.02702 (18)	−0.00772 (17)	0.00296 (17)
S1	0.0513 (2)	0.0616 (3)	0.0474 (2)	−0.0268 (2)	−0.01145 (18)	0.00232 (19)

### Geometric parameters (Å, °)

**Table:**

C1—C2	1.391 (2)	C12'—C13'	1.373 (3)
C1—C6	1.406 (2)	C12'—H12'	0.9300
C1—N1	1.414 (2)	C12—C13	1.379 (3)
C1'—C6'	1.397 (2)	C12—H12	0.9300
C1'—C2'	1.397 (2)	C13—C14	1.377 (3)
C1'—N1'	1.4150 (18)	C13—H13	0.9300
C2'—C3'	1.378 (3)	C13'—C14'	1.386 (3)
C2'—H2'	0.9300	C13'—H13'	0.9300
C2—C3	1.378 (3)	C14'—C15'	1.372 (2)
C2—H2	0.9300	C14'—H14'	0.9300
C3—C4	1.383 (3)	C14—C15	1.387 (3)
C3—H3	0.9300	C14—H14	0.9300
C3'—C4'	1.393 (3)	C15—H15	0.9300
C3'—H3'	0.9300	C15'—H15'	0.9300
C4'—C5'	1.370 (3)	C16—C17	1.374 (2)
C4'—H4'	0.9300	C16—C21	1.381 (2)
C4—C5	1.372 (3)	C16—S1	1.7551 (16)
C4—H4	0.9300	C16'—C17'	1.384 (2)
C5—C6	1.398 (2)	C16'—C21'	1.384 (2)
C5—H5	0.9300	C16'—S1'	1.7563 (16)
C5'—C6'	1.404 (2)	C17'—C18'	1.383 (3)
C5'—H5'	0.9300	C17'—H17'	0.9300
C6—C7	1.425 (2)	C17—C18	1.385 (3)
C6'—C7'	1.423 (2)	C17—H17	0.9300
C7—C8	1.350 (2)	C18'—C19'	1.375 (3)
C7—H7	0.9300	C18'—H18'	0.9300
C7'—C8'	1.352 (2)	C18—C19	1.374 (3)
C7'—H7'	0.9300	C18—H18	0.9300
C8'—N1'	1.4179 (18)	C19—C20	1.376 (3)
C8'—C9'	1.477 (2)	C19—H19	0.9300
C8—N1	1.416 (2)	C19'—C20'	1.380 (3)
C8—C9	1.479 (2)	C19'—H19'	0.9300
C9'—O3'	1.2158 (19)	C20—C21	1.376 (3)
C9'—C10'	1.486 (2)	C20—H20	0.9300
C9—O3	1.216 (2)	C20'—C21'	1.379 (3)
C9—C10	1.487 (2)	C20'—H20'	0.9300
C10'—C11'	1.389 (2)	C21'—H21'	0.9300
C10'—C15'	1.390 (2)	C21—H21	0.9300
C10—C11	1.388 (2)	N1—S1	1.6734 (13)
C10—C15	1.393 (2)	N1'—S1'	1.6761 (12)
C11—C12	1.375 (2)	O1'—S1'	1.4254 (12)
C11—H11	0.9300	O1—S1	1.4264 (14)
C11'—C12'	1.388 (2)	O2'—S1'	1.4197 (13)
C11'—H11'	0.9300	O2—S1	1.4217 (13)
			
C2—C1—C6	121.55 (16)	C14—C13—C12	120.26 (18)
C2—C1—N1	131.80 (16)	C14—C13—H13	119.9
C6—C1—N1	106.64 (14)	C12—C13—H13	119.9
C6'—C1'—C2'	121.26 (14)	C12'—C13'—C14'	120.30 (16)
C6'—C1'—N1'	107.10 (13)	C12'—C13'—H13'	119.8
C2'—C1'—N1'	131.59 (14)	C14'—C13'—H13'	119.8
C3'—C2'—C1'	117.05 (17)	C15'—C14'—C13'	119.78 (16)
C3'—C2'—H2'	121.5	C15'—C14'—H14'	120.1
C1'—C2'—H2'	121.5	C13'—C14'—H14'	120.1
C3—C2—C1	117.18 (19)	C13—C14—C15	120.16 (19)
C3—C2—H2	121.4	C13—C14—H14	119.9
C1—C2—H2	121.4	C15—C14—H14	119.9
C2—C3—C4	122.13 (19)	C14—C15—C10	119.49 (18)
C2—C3—H3	118.9	C14—C15—H15	120.3
C4—C3—H3	118.9	C10—C15—H15	120.3
C2'—C3'—C4'	122.34 (17)	C14'—C15'—C10'	120.45 (16)
C2'—C3'—H3'	118.8	C14'—C15'—H15'	119.8
C4'—C3'—H3'	118.8	C10'—C15'—H15'	119.8
C5'—C4'—C3'	120.69 (16)	C17—C16—C21	121.50 (16)
C5'—C4'—H4'	119.7	C17—C16—S1	119.44 (13)
C3'—C4'—H4'	119.7	C21—C16—S1	118.96 (13)
C5—C4—C3	120.89 (18)	C17'—C16'—C21'	121.27 (15)
C5—C4—H4	119.6	C17'—C16'—S1'	119.65 (13)
C3—C4—H4	119.6	C21'—C16'—S1'	118.99 (12)
C4—C5—C6	118.82 (18)	C18'—C17'—C16'	118.61 (16)
C4—C5—H5	120.6	C18'—C17'—H17'	120.7
C6—C5—H5	120.6	C16'—C17'—H17'	120.7
C4'—C5'—C6'	118.42 (17)	C16—C17—C18	118.52 (18)
C4'—C5'—H5'	120.8	C16—C17—H17	120.7
C6'—C5'—H5'	120.8	C18—C17—H17	120.7
C5—C6—C1	119.41 (16)	C19'—C18'—C17'	120.57 (17)
C5—C6—C7	132.82 (17)	C19'—C18'—H18'	119.7
C1—C6—C7	107.72 (14)	C17'—C18'—H18'	119.7
C1'—C6'—C5'	120.22 (15)	C19—C18—C17	120.33 (19)
C1'—C6'—C7'	107.86 (13)	C19—C18—H18	119.8
C5'—C6'—C7'	131.83 (16)	C17—C18—H18	119.8
C8—C7—C6	108.95 (15)	C18—C19—C20	120.59 (18)
C8—C7—H7	125.5	C18—C19—H19	119.7
C6—C7—H7	125.5	C20—C19—H19	119.7
C8'—C7'—C6'	108.73 (14)	C18'—C19'—C20'	120.29 (17)
C8'—C7'—H7'	125.6	C18'—C19'—H19'	119.9
C6'—C7'—H7'	125.6	C20'—C19'—H19'	119.9
C7'—C8'—N1'	108.63 (13)	C21—C20—C19	119.68 (18)
C7'—C8'—C9'	126.66 (14)	C21—C20—H20	120.2
N1'—C8'—C9'	123.14 (13)	C19—C20—H20	120.2
C7—C8—N1	108.47 (14)	C21'—C20'—C19'	120.09 (17)
C7—C8—C9	126.76 (15)	C21'—C20'—H20'	120.0
N1—C8—C9	123.09 (14)	C19'—C20'—H20'	120.0
O3'—C9'—C8'	120.50 (14)	C20'—C21'—C16'	119.15 (15)
O3'—C9'—C10'	121.06 (14)	C20'—C21'—H21'	120.4
C8'—C9'—C10'	118.33 (13)	C16'—C21'—H21'	120.4
O3—C9—C8	120.57 (15)	C20—C21—C16	119.37 (17)
O3—C9—C10	121.10 (15)	C20—C21—H21	120.3
C8—C9—C10	118.26 (14)	C16—C21—H21	120.3
C11'—C10'—C15'	119.57 (14)	C1—N1—C8	108.21 (13)
C11'—C10'—C9'	122.77 (14)	C1—N1—S1	126.37 (11)
C15'—C10'—C9'	117.61 (14)	C8—N1—S1	124.59 (11)
C11—C10—C15	119.75 (16)	C1'—N1'—C8'	107.66 (12)
C11—C10—C9	118.40 (15)	C1'—N1'—S1'	126.32 (10)
C15—C10—C9	121.76 (15)	C8'—N1'—S1'	124.95 (10)
C12—C11—C10	120.12 (17)	O2'—S1'—O1'	119.73 (8)
C12—C11—H11	119.9	O2'—S1'—N1'	107.50 (7)
C10—C11—H11	119.9	O1'—S1'—N1'	104.83 (7)
C12'—C11'—C10'	119.56 (16)	O2'—S1'—C16'	110.44 (8)
C12'—C11'—H11'	120.2	O1'—S1'—C16'	107.87 (8)
C10'—C11'—H11'	120.2	N1'—S1'—C16'	105.46 (7)
C13'—C12'—C11'	120.25 (18)	O2—S1—O1	119.56 (9)
C13'—C12'—H12'	119.9	O2—S1—N1	107.30 (7)
C11'—C12'—H12'	119.9	O1—S1—N1	105.18 (7)
C11—C12—C13	120.17 (18)	O2—S1—C16	110.09 (8)
C11—C12—H12	119.9	O1—S1—C16	108.48 (8)
C13—C12—H12	119.9	N1—S1—C16	105.20 (7)
			
C6'—C1'—C2'—C3'	−1.4 (2)	C13'—C14'—C15'—C10'	−3.2 (2)
N1'—C1'—C2'—C3'	175.84 (16)	C11'—C10'—C15'—C14'	2.0 (2)
C6—C1—C2—C3	1.7 (3)	C9'—C10'—C15'—C14'	179.39 (14)
N1—C1—C2—C3	−176.50 (17)	C21'—C16'—C17'—C18'	−0.8 (2)
C1—C2—C3—C4	−0.6 (3)	S1'—C16'—C17'—C18'	−177.48 (14)
C1'—C2'—C3'—C4'	0.1 (3)	C21—C16—C17—C18	0.8 (3)
C2'—C3'—C4'—C5'	0.6 (3)	S1—C16—C17—C18	177.02 (16)
C2—C3—C4—C5	−0.5 (3)	C16'—C17'—C18'—C19'	0.2 (3)
C3—C4—C5—C6	0.4 (3)	C16—C17—C18—C19	−0.1 (3)
C3'—C4'—C5'—C6'	0.1 (3)	C17—C18—C19—C20	−0.6 (3)
C4—C5—C6—C1	0.7 (3)	C17'—C18'—C19'—C20'	0.5 (3)
C4—C5—C6—C7	177.74 (18)	C18—C19—C20—C21	0.5 (3)
C2—C1—C6—C5	−1.8 (2)	C18'—C19'—C20'—C21'	−0.5 (3)
N1—C1—C6—C5	176.81 (15)	C19'—C20'—C21'—C16'	−0.2 (3)
C2—C1—C6—C7	−179.50 (16)	C17'—C16'—C21'—C20'	0.8 (2)
N1—C1—C6—C7	−0.91 (17)	S1'—C16'—C21'—C20'	177.51 (13)
C2'—C1'—C6'—C5'	2.1 (2)	C19—C20—C21—C16	0.2 (3)
N1'—C1'—C6'—C5'	−175.76 (14)	C17—C16—C21—C20	−0.9 (3)
C2'—C1'—C6'—C7'	179.13 (15)	S1—C16—C21—C20	−177.14 (14)
N1'—C1'—C6'—C7'	1.29 (17)	C2—C1—N1—C8	178.72 (17)
C4'—C5'—C6'—C1'	−1.4 (2)	C6—C1—N1—C8	0.33 (17)
C4'—C5'—C6'—C7'	−177.59 (17)	C2—C1—N1—S1	−11.4 (3)
C5—C6—C7—C8	−176.10 (18)	C6—C1—N1—S1	170.23 (11)
C1—C6—C7—C8	1.19 (19)	C7—C8—N1—C1	0.41 (18)
C1'—C6'—C7'—C8'	−1.27 (18)	C9—C8—N1—C1	−165.74 (14)
C5'—C6'—C7'—C8'	175.31 (17)	C7—C8—N1—S1	−169.71 (12)
C6'—C7'—C8'—N1'	0.73 (18)	C9—C8—N1—S1	24.1 (2)
C6'—C7'—C8'—C9'	−165.23 (15)	C6'—C1'—N1'—C8'	−0.86 (16)
C6—C7—C8—N1	−0.98 (18)	C2'—C1'—N1'—C8'	−178.39 (16)
C6—C7—C8—C9	164.52 (15)	C6'—C1'—N1'—S1'	−169.40 (11)
C7'—C8'—C9'—O3'	139.58 (18)	C2'—C1'—N1'—S1'	13.1 (2)
N1'—C8'—C9'—O3'	−24.5 (2)	C7'—C8'—N1'—C1'	0.08 (17)
C7'—C8'—C9'—C10'	−36.7 (2)	C9'—C8'—N1'—C1'	166.64 (14)
N1'—C8'—C9'—C10'	159.25 (14)	C7'—C8'—N1'—S1'	168.82 (12)
C7—C8—C9—O3	−135.37 (19)	C9'—C8'—N1'—S1'	−24.6 (2)
N1—C8—C9—O3	28.2 (2)	C1'—N1'—S1'—O2'	137.76 (13)
C7—C8—C9—C10	41.5 (2)	C8'—N1'—S1'—O2'	−28.89 (15)
N1—C8—C9—C10	−154.98 (15)	C1'—N1'—S1'—O1'	9.35 (15)
O3'—C9'—C10'—C11'	149.60 (16)	C8'—N1'—S1'—O1'	−157.30 (13)
C8'—C9'—C10'—C11'	−34.2 (2)	C1'—N1'—S1'—C16'	−104.39 (13)
O3'—C9'—C10'—C15'	−27.7 (2)	C8'—N1'—S1'—C16'	88.95 (14)
C8'—C9'—C10'—C15'	148.56 (14)	C17'—C16'—S1'—O2'	−8.10 (15)
O3—C9—C10—C11	27.8 (2)	C21'—C16'—S1'—O2'	175.18 (12)
C8—C9—C10—C11	−148.99 (15)	C17'—C16'—S1'—O1'	124.45 (13)
O3—C9—C10—C15	−148.71 (18)	C21'—C16'—S1'—O1'	−52.28 (14)
C8—C9—C10—C15	34.5 (2)	C17'—C16'—S1'—N1'	−123.95 (13)
C15—C10—C11—C12	−1.1 (3)	C21'—C16'—S1'—N1'	59.33 (13)
C9—C10—C11—C12	−177.72 (16)	C1—N1—S1—O2	−140.16 (13)
C15'—C10'—C11'—C12'	0.8 (2)	C8—N1—S1—O2	28.17 (15)
C9'—C10'—C11'—C12'	−176.44 (15)	C1—N1—S1—O1	−11.85 (15)
C10'—C11'—C12'—C13'	−2.4 (3)	C8—N1—S1—O1	156.48 (13)
C10—C11—C12—C13	2.1 (3)	C1—N1—S1—C16	102.62 (14)
C11—C12—C13—C14	−1.4 (3)	C8—N1—S1—C16	−89.06 (14)
C11'—C12'—C13'—C14'	1.2 (3)	C17—C16—S1—O2	13.85 (17)
C12'—C13'—C14'—C15'	1.6 (3)	C21—C16—S1—O2	−169.87 (14)
C12—C13—C14—C15	−0.4 (3)	C17—C16—S1—O1	−118.69 (15)
C13—C14—C15—C10	1.4 (3)	C21—C16—S1—O1	57.58 (15)
C11—C10—C15—C14	−0.6 (3)	C17—C16—S1—N1	129.16 (15)
C9—C10—C15—C14	175.87 (18)	C21—C16—S1—N1	−54.56 (15)

## References

[bb1] Allen, F. H., Kennard, O., Watson, D. G., Brammer, L., Orpen, A. G. & Taylor, R. (1987). *J. Chem. Soc. Perkin Trans. 2*, pp. S1–19.

[bb2] Beddoes, R. L., Dalton, L., Joule, T. A., Mills, O. S., Street, J. D. & Watt, C. I. F. (1986). *J. Chem. Soc. Perkin Trans. 2*, pp. 787–797.

[bb3] Bruker (2007). *APEX2* and *SAINT* Bruker AXS Inc., Madison Wisconsin, USA.

[bb4] Chakkaravarthi, G., Panchatcharam, R., Dhayalan, V., Mohanakrishnan, A. K. & Manivannan, V. (2010). *Acta Cryst.* E**66**, o2957. 10.1107/S160053681004198X PMC3009294 21589125

[bb5] Farrugia, L. J. (1997). *J. Appl. Cryst.* **30**, 565.

[bb6] Kavitha, T., Thenmozhi, M., Dhayalan, V., Mohanakrishnan, A. K. & Ponnuswamy, M. N. (2010). *Acta Cryst.* E**66**, o1071. 10.1107/S160053681001247X PMC2979030 21579126

[bb7] Ma, C., Liu, X., Li, X., Flippen-Anderson, J., Yu, S. & Cook, J. M. (2001). *J. Org. Chem.* **66**, 4525–4542. 10.1021/jo001679s 11421771

[bb8] Sheldrick, G. M. (1996). *SADABS* University of Göttingen, Germany.

[bb9] Sheldrick, G. M. (2008). *Acta Cryst.* A**64**, 112–122. 10.1107/S0108767307043930 18156677

[bb10] Spek, A. L. (2009). *Acta Cryst.* D**65**, 148–155. 10.1107/S090744490804362X PMC2631630 19171970

[bb11] Williams, T. M., Ciccarone, T. M., MacTough, S. C., Rooney, C. S., Balani, S. K., Condra, J. H., Emini, E. A., Goldman, M. E., Greenlee, W. J. & Kauffman, L. R. (1993). *J. Med. Chem.* **36**, 1291–1294. 10.1021/jm00061a022 7683725

[bb12] Zhao, S., Liao, X. & Cook, J. M. (2002). *Org. Lett.* **4**, 687–690. 10.1021/ol010222h 11869102

[bb13] Zhou, H., Liao, X., Yin, W., Ma, J. & Cook, J. M. (2006). *J. Org. Chem.* **71**, 251–259. 10.1021/jo052081t 16388644

